# Alterations in Implantation Genes and Dendritic Cells in Endometrial Samples After Antibiotic Treatment

**DOI:** 10.3390/jcm14030834

**Published:** 2025-01-27

**Authors:** Baraa Darawshe, Shay Hantisteanu, Shilhav Meisel Sharon, Gabriel Groisman, Sergio Haimovich, Einat Shalom-Paz

**Affiliations:** 1 IVF Unit, Hillel Yaffe Medical Center, Hadera 38100, Israelshilhav.ms@gmail.com (S.M.S.); groisman@hy.health.gov.il (G.G.); sagih@hy.health.gov.il (S.H.); einatshalompaz@gmail.com (E.S.-P.); 2 Rappaport Faculty of Medicine, The Technion–Israel Institute of Technology, Haifa 32000, Israel

**Keywords:** chronic endometritis, recurrent implantation failure, insulin-like growth factor-1, *IGF-1*, Homeobox A10, *HOXA10*, immunohistochemistry

## Abstract

**Background/Objectives:** This retrospective study assessed the impact of chronic endometritis (CE) on the expression of implantation genes *HOXA10* (Homeobox A10) and *IGF-1* (insulin-like growth factor 1), and on dendritic cells before and after antibiotic treatment, as well as on clinical reproductive outcomes. **Methods:** The study was conducted from 2021 to 2022. Ten assisted reproductive technology patients who underwent an endometrial biopsy before antibiotic treatment, confirming the diagnosis of CE, and a second biopsy after completing a course of 100 mg doxycycline twice daily for 14 days were included. Paraffin-embedded endometrial samples from these patients were obtained from the pathology department. The samples were evaluated for quantifying implantation genes *HOXA10* and *IGF-1* using RT-PCR, and for identification of dendritic cells using immunohistochemical staining of CD141. Conceptions and live births were also evaluated. **Results:** Endometrial expression of *HOXA10* and *IGF-1* genes was significantly elevated after antibiotic treatment and expression of dendritic cells CD141 was decreased. Eight of the ten patients required second-line antibiotic treatment due to persistent CE. Six patients conceived and delivered. **Conclusions:** CE is reversible with antibiotic treatment that resulted in improvements in implantation genes and clinical results. A high proportion of patients required broader spectrum antibiotic treatment.

## 1. Introduction

Chronic endometritis (CE) is characterized by persistent inflammation of the endometrium. Inflammation alters endometrial immune components, which may impair endometrial function and result in reduced embryo receptivity [1]. Chronic endometritis is often asymptomatic, typically diagnosed during evaluation for secondary amenorrhea or infertility [2]. It may also present with subtle symptoms, including dysfunctional uterine bleeding, pelvic discomfort, or leukorrhea. Therefore, it may be overlooked in clinical practice [3]. The prevalence of chronic endometritis is frequently underestimated due to the challenges in diagnosing the condition. Its prevalence varies between 0.2% and 46%, depending on factors such as the patient population and biopsy techniques used [2].

It is well-established that the success of embryo implantation depends on the complex communication of embryo quality, uterine integrity, and endometrial receptivity. Any endometrial condition, whether resulting from hormonal imbalances or inflammation, can affect endometrial receptivity and contribute to infertility [4].

Repeated implantation failure (RIF) is defined after failure of three embryo transfer cycles in which high-quality embryos were transferred. Recent data increasingly suggest that chronic endometritis (CE) may be a potential cause of RIF, early miscarriage, recurrent pregnancy loss (RPL), and certain obstetric complications [5]. Evidence shows that 30% of RIF cases involved CE. Patients with CE have lower implantation rates and recurrent miscarriages [6,7]. It has been demonstrated that antibiotic treatment of patients with CE improved pregnancy outcomes in in vitro fertilization (IVF) [4]. It is estimated that up to 56.8% of women with infertility are diagnosed with CE, a prevalence that is twice as high as that seen in fertile women and greater than that reported in the general population [8]. The diagnostic methods for CE commonly used in routine practice are relatively simple. Endometrial sampling is typically carried out using hysteroscopy. To date, internationally recognized diagnostic criteria have not been established for chronic endometritis and the criteria proposed in various studies are often selected arbitrarily [5,8].

This study evaluated the impact of CE on implantation before and after antibiotic treatment. We used three markers: *HOXA10* (Homeobox A10, *IGF-1* (insulin-like growth factor 1) and dendritic cells (DCs) in the endometrium as implantation markers.

*HOXA10* and *IGF-1* gene expressions are known to be correlated with endometrial quality and implantation. DC are specialized, highly potent antigen-presenting cells that initiate and regulate innate and adaptive immune responses, in our case, the response to CE.

This study evaluated expression levels of the key implantation genes *IGF-1* and *HOXA10* and the presence of DCs before and after antibiotic treatment, in the endometria of patients diagnosed with CE undergoing assisted reproductive technology (ART) treatments.

## 2. Materials and Methods

### 2.1. Patients

Study participants were 21- to 44-year-old women with infertility, undergoing IVF treatment from 2019 to 2021, in the Department of Obstetrics and Gynecology at Hillel Yaffe Medical Center, Hadera, Israel, who were referred for hysteroscopy. Patients with hysteroscopic signs of CE confirmed by histology were enrolled in the study.

### 2.2. Exclusion Criteria

Patients included in this study were diagnosed with CE according to hysteroscopy sampling and pathology staining and/or RIF or RPL. RIF was diagnosed when more than three high-quality embryos were transferred with failure to achieve pregnancy. RPL was diagnosed when more than three miscarriages occurred.

Exclusion criteria were women older than 44 years of age, other causes that may contribute to RIF or RPL, such as genetic problems, dysmorphic uterus, thrombotic disease, antiphospholipid antibodies or thrombophilia, as well as severe oligo-astheno-teratozoospermia or azoospermia.

The clinic protocol is to refer patients with more than two implantation failures for hysteroscopy. Hysteroscopy was scheduled during the early follicular phase, just after cessation of menstruation. During hysteroscopy, endometrial samples were obtained for histological diagnosis only from patients suspected to have CE. Hysteroscopic signs of CE included focal or global edema, strawberry aspect, and micropolyps. In this study, CE was diagnosed based on >10 MUM-1 (multiple myeloma oncogene 1) cells per 10 high-power fields (HPFs) in the endometrial samples. Samples were then paraffinized. Based on the clinical signs seen on hysteroscopy, patients were treated with a course of doxycycline 100 mg twice daily for 14 days, according to the clinic protocol, and at that point were included in the study. Paraffinized samples from patients with CE were evaluated retrospectively.

To evaluate the effect of antibiotic treatment, a second endometrial evaluation and biopsy were taken 1 month later, using a Pipelle endometrial suction curette (PRODiMED, Neuilly-en-Thelle, France). The second sample was also evaluated for MUM-1 cells and stored in paraffin blocks. Women whose samples were still positive for MUM-1 received a second line of antibiotic treatment with ciprofloxacin 500 mg twice daily and metronidazole 500 mg three times a day for 14 days.

All paraffin-embedded samples that were positive for MUM-1 and the second biopsy of those patients were included, using the following methods:

1. The implantation genes *HOXA10* and *IGF-1* were quantified using RT-PCR in samples obtained before and after antibiotic treatment.
2. DCs were identified using immunohistochemical (IHC) staining of CD141 and *IGF-1* before and after antibiotic treatment.

### 2.3. Deparaffinization

Total RNA was extracted from paraffin blocks using RNeasy FFPE kit (Qiagen Biotech, Hilden, Germany, Cat. No. 73504). Endometrial sample blocks from women diagnosed with CE were cut into 5 μm sections, deparaffinized on heat blocks for 15 min at 56 °C and 80 °C, using a deparaffination solution (Zotal, ZR-D3067-1-20, 4 Habarzel St. Tel Aviv 69710, Israel).

Buffer PKD (Proteinase K Digestion) was added and mixed by vortexing. Proteinase K was added to the mix and centrifuged for 1 min at 11,000× *g* (10,000 rpm). The mixture was incubated on ice for 3 min and afterwards centrifuged for 15 min. Buffer red blood cells (RBCs) and ethanol 100% were added to adjust binding conditions.

The mixture was then transferred to an RNeasy MinElute spin column placed inside a collection tube. Buffer RPE and RNase free water were added to the spin column membrane and underwent repeated centrifugation to elute the RNA.

### 2.4. Quantitative Real-Time PCR

The molecular structures of implantation genes *HOXA10* and *IGF-1* were identified using RT-PCR and specific primers, total RNA was extracted from deparaffinized samples using RNeasy FFPE kit (Qiagen Biotech, Cat. No. 73504), according to the manufacturer’s protocol, as detailed in Section 2.3, *Deparaffinization*. RNA from the endometrial samples was used for each reverse transcription reaction. RNA was polyA-primed by incubation with Oligo dT and dNTPs and denatured at 65 °C. This was followed by the addition of a master mix of 80 μL total volume containing 1× SuperScript buffer, 10 mM DTT, 5 mM magnesium chloride, RNaseOUT, and SuperScript III reverse transcriptase (Invitrogen, Waltham, MA, USA). Reactions were then incubated at 50 °C for 50 min. Reactions were terminated by incubation at 85 °C for 5 min. Three μL of cDNA from the above reaction were used as templates for the first round of PCR using the primers listed in Table 1. PCR master mixes included DMSO. PCR reactions were performed using the program in Table 2. After quantifying the expression of implantation genes in each sample, the two samples were compared.

### 2.5. Immunohistochemistry

After deparaffinization, immunofluorescence was conducted on slides of endometrial samples with CE as follows: 5 μm of paraffinized endometrial sample sections were cut on coated slides. Sections were deparaffinized in xylene and passed through 3 × 10 min 99% ethanol and 2 × 10 min 96% ethanol. Endogenous peroxidase activity was then blocked with 20% horse serum (inactivated 55°) for 30 min. Rehydration was completed by rinsing in ethanol and ultrapure water. Antigens were retrieved using a 10 mM citrate buffer in a microwave for 20 min. Primary antibody was used for staining CD141 at a dilution of 1:50. Anti-Rabbit Cy3 (Jackson labs, Bar Harbor, ME, USA, Cat. #711165152) was used as a secondary antibody at a dilution of 1:200. Slides were then counterstained with Prolong DAPI staining, (ThermoFisher, Waltham, MA, USA) and mounted. Images were taken using ImageJ 1.54g software.

## 3. Results

**Figure 1:**
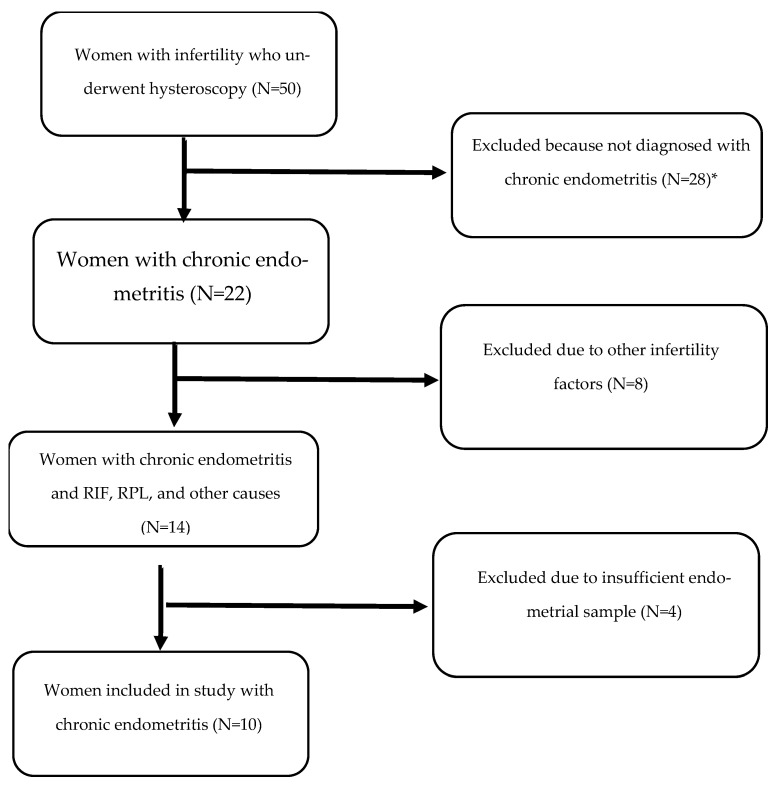
Flow diagram of study population. * Did not meet criteria for chronic endometritis (>10 MUM-1 cells/10 HPF). RIF, repeated implantation failure.

Paraffin embedded tissues of 50 patients were included in the study. Only patients with CE diagnosed by pathology samples with positive staining of >10 MUM-1 cells/10 HPF and no uterine abnormality were included. Excluded were 28 patients with no sign of CE, 8 due to other infertility reasons (age above age 44, uterine malformation, male factor infertility, thrombophilia or other genetic problems) and 4 due to insufficient endometrial samples (Figure 1).

A total of 10 patients were included. Their mean age was 35.1 years, mean duration of infertility was 3.4 years, and 60% were nulliparous. Seven were healthy without known disease, whereas one had multiple sclerosis in remission, one had diabetes, and one had ankylosing spondylitis (Table 3).

### 3.1. Real-Time PCR Results

**Figure 2:**
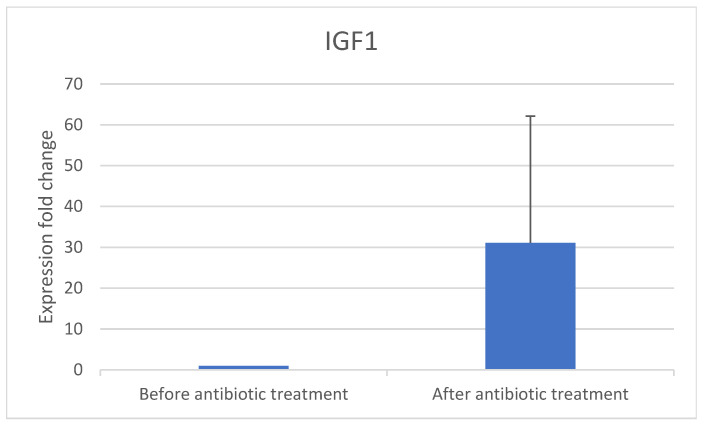
*IGF-1* gene expression fold change before and after antibiotic treatment.

**Figure 3:**
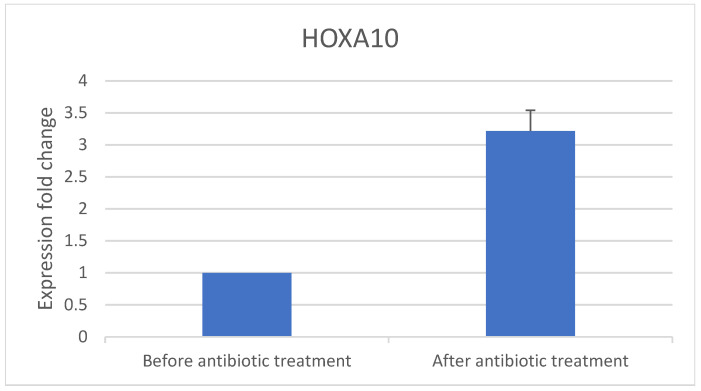
*HOXA10* gene expression fold change before and after antibiotic treatment.

PCR was used to examine the 10 samples for expression of the implantation genes *IGF-1* (Figure 2) and *HOXA10* (Figure 3) before and after antibiotic treatment.

Implantation gene expression increased after antibiotic treatment: *IGF-1* increased 31-fold and *HOXA10* increased 3.5-fold. Eight patients conceived via IVF after completing antibiotic treatment.

### 3.2. Immunohistochemistry Results

**Figure 4:**
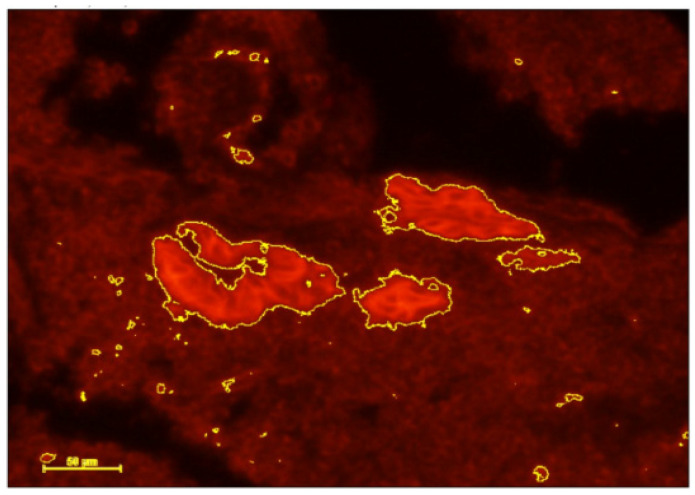
Endometrial sample with CD141 staining (highlighted area) before antibiotic treatment.

**Figure 5:**
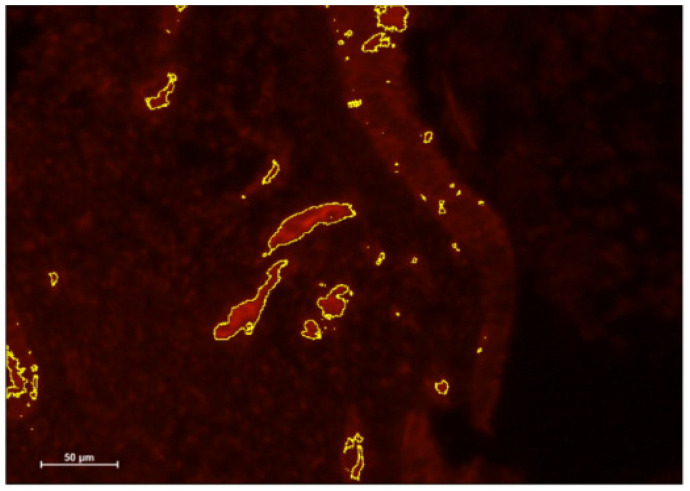
Endometrial sample with CD141 staining (highlighted area) after antibiotic treatment.

The results of the IHC staining showed that the inflammation markers decreased from before antibiotic treatment (Figure 4) to after (Figure 5). The results indicate that the intervention decreased DC staining.

## 4. Discussion

This preliminary study aimed to demonstrate a proof of concept regarding the impact of antibiotic treatment on women undergoing ART treatments, who were diagnosed with CE based on endometrial signs during hysteroscopy and samples positive for MUM-1.

Inflammation markers of DCs decreased in women with CE following antibiotic treatment (Figure 4 and Figure 5) and expression of implantation genes *HOXA10* and *IGF-1* increased (Figure 2 and Figure 3). Pregnancy outcomes were favorable after antibiotic treatment, demonstrating an 80% pregnancy rate, with 60% live birth rate (Table 3).

The standard criterion for the diagnosis of CE is fluid hysteroscopy during the proliferative phase of the menstrual cycle. Stromal edema, focal diffuse epithelial hyperemia, and micropolyps may be visualized macroscopically [3,9]. Concomitantly, endometrial biopsy is taken for histopathology and staining to determine the presence of CD138 plasma cells. Microbial cultures may reveal microbial origins of the inflammation; thus, allowing targeted antibiotic therapy. However, the yield of this treatment is limited [10,11]. Although CE has various etiologies, an infectious etiology is considered the prime cause because in many cases, it regresses following antibiotic treatment. However, resistance to antibiotics suggests other etiologies, such as an autoimmune process that may cause endometrial changes that alter normal endometrial receptivity [12]. CE can alter the expression of endometrial cytokines, growth factors, and apoptotic proteins [13]. In vitro studies demonstrate that bacterial lipopolysaccharides can induce the expression of genes that result in an abnormal immune response with migration of circulating B lymphocytes towards the endometrial stroma and plasma cells, which could adversely affect implantation [8].

Currently, diagnosis of CE is based on IHC staining of CD138 or MUM-1 cells. Plasma cells in the endometrium are an indication of CE and are considered a more accurate and sensitive method for diagnosing the condition [14] compared to other methods. Nonetheless, according to previous reports [7,9], diagnosing CE according to anti-CD138 staining is inconsistent, which is why we used MUM-1 as a marker for diagnosing CE in this study.

DCs are specialized, highly potent antigen-presenting cells that initiate and regulate innate and adaptive immune responses. DCs can integrate into blood vessel endothelial cells and stimulate angiogenesis by secreting proangiogenic factors and cytokines. Previous studies have shown that DCs are prominent in the endometrium of healthy, fertile controls compared with patients with recurrent miscarriages and RIF [15]. A successful pregnancy requires the maternal immune system to tolerate the semi-allogeneic fetus. Indeed, DCs are recruited to the uterus prior to implantation and modulate the cytokine profile at the feto-maternal interface. Adequate inflammation induced by DCs is required to ensure successful implantation and prevent first trimester miscarriage [16].

Insulin-like growth factors are important players in endometrial proliferation, differentiation, and in embryo-endometrial interactions. To date, information on the biochemical and paracrine alterations in the endometrium of women diagnosed with CE is scarce [17]. As described previously [15,18], women with CE have significant alterations in the expression profile of genes involved in growth factor synthesis and in cellular proliferation. Expression of the insulin-like growth factor binding protein-1 (*IGFBP-1*) gene was significantly increased in the endometrium of women with CE, while *IGF-1* was reduced. Proper *IGF* levels are required for successful embryonic and placental development [18]. IGF-1 plays an important role in the relationship between the embryo and the endometrium, mediating the effects of estrogen on endometrial proliferation in the proliferative phase. It is well-established that adequate IGF-1 levels are essential for successful embryonic and placental development. As a result, the downregulation of IGF-1 could be linked to the poor endometrial quality and infertility seen in women with CE, and may explain the histological changes frequently present in these women, such as the presence of polyps [8,15,19].

Additionally, Nazarenko et al. described alterations in the expression of genes in the endometrium of women with RIF and RPL [20], which are considered critical for implantation. Previous studies in mice yielded substantial information regarding the role of individual genes in the process of uterine receptivity and blastocyst implantation, including growth factors, cytokines, and transcription factors. Among these genes are *HOXA10*.

In accordance with our study, *HOXA10* and *IGF-1* gene expressions are known to be correlated with endometrial quality and implantation. *HOXA10* is among a variety of genes that are expressed in the endometrial glands and stroma of the human uterus throughout the menstrual cycle. The expression of these genes increases dramatically at the time of implantation, suggesting that *HOXA10* is one of many genes that play a role in human implantation [19]. To the best of our knowledge, this study was the first to evaluate *HOXA10* gene expression in correlation with CE and antibiotic treatment.

This study showed that reduced *IGF-1* expression, alongside *HOXA10* in women with CE suggests a detrimental condition for implantation. Our results agree with those of Di Pietro et al. [15] who demonstrated different expression of 25 inflammatory responses, proliferation, and apoptosis genes in the endometrium of women with or without CE, including lower expression of *IGF-1*. Park et al. [21] showed that decreased *IGF-1* gene expression in CE, along with alterations in other gene expressions create unfavorable conditions for implantation and embryo development. Our results support those of Li et al. [14] as well, who reported that CE is associated with increased infiltration of immune cells in the endometrium, which might have a role in diminishing endometrial receptivity. After antibiotic treatment, a significant decrease in uterine immune cells was observed.

The present study resulted in an 80% pregnancy rate after antibiotic treatment and a 60% live birth rate. The positive impact of antibiotic treatment on endometrial receptivity was also shown in studies by Vaduva et al. [4]. Kitaya et al. [22] and Cicinelli et al. [23] reported that women with CE, cured after antibiotic therapy, had higher pregnancy and live birth rates compared to women with persistent CE. For this reason, it is recommended to administer antibiotic treatment in all cases where endometrial inflammation is observed through direct examination and histological analysis.

In the current study, 8 of 10 patients received a second course of antibiotics with ciprofloxacin and metronidazole; 7 due to persistent markers of CE at the follow-up visit and another due to doxycycline allergy.

The diagnostic criteria for CE presented in the literature are inconsistent. The threshold of plasma cells in the endometrium varies. Li et al. [14] refer to more than five plasma cells/HPF. Hirata et al. [24] investigated the pregnancy, live birth, and miscarriage rates among women with and without CE, divided into four groups according to the number of plasma cells in 10 HPF: ≥1, ≥2, ≥3, or ≥5. The current study included patients with ≥10 plasma cells per 10 HPF, which may indicate the severity of CE. This may also explain the persistent CE and the need for a second line of antibiotic treatment.

The pregnancy rate in this study was 80%, where 8 of the 10 women had a positive beta HCG test, of which one was a chemical pregnancy and another an ectopic pregnancy. The live birth rate was 60%, where 6 patients had a successful pregnancy and delivery. In a prospective study by Kitaya et al. [22], endometrial biopsy samples were obtained from infertile women with RIF and CE. Following antibiotic administration, the reproductive rate was evaluated, among other parameters. They found that following antibiotic treatment, the live birth rate in three cumulative embryo transfer cycles among the women with RIF and cured CE was significantly higher than in the RIF group without CE. In addition, Espinós et al. [8] showed that patients with CE and a history of RIF who respond to antibiotic treatment tend to have improved pregnancy and implantation rates, as well as higher live birth rates, compared to those with recurrent CE not responsive to antibiotic treatment. These results emphasized the effectiveness of antibiotic treatment among patients diagnosed with CE whose reproductive outcomes subsequently improved. The current study provided additional evidence that antibiotic treatment given to women with RIF and CE positively affected reproductive results.

This preliminary study had some limitations. The sample size was small. The use of paraffin-embedded samples and prolonged storage may impact the expression of different genes or molecular markers. Moreover, the study population was heterogeneous, with different infertility diagnoses.

Despite these limitations, this study provides a proof of concept that antibiotic treatment may positively affect fertility in women with CE. This underscores that CE is reversible, leading to favorable reproductive outcomes after antibiotic treatment. Moreover, we followed our patients through conception, pregnancy, and delivery. Therefore, better characterization of CE may enable caregivers to recognize these patients and provide appropriate treatment sooner.

## 5. Conclusions

The results of this study confirm our initial hypothesis, that antibiotic treatment increases the expression of *IGF-1* and *HOXA10* genes in endometrial samples, with positive effects on fertility. Furthermore, some patients may require a broader spectrum of antibiotic treatment, potentially related to the severity of CE. Further studies are needed to validate the diagnosis of CE, assess its severity, and tailor antibiotic treatment accordingly.

## Figures and Tables

**Table 1:** Primers used for the PCR.

Oligo Name	Sequence (5′-3′)
HOXA 10-F	CAACTGGCTCACGGCAAAGA
HOXA 10-R	TTCAGTTTCATCCTGCGGTTC
IGF1-F	GTGGAGACAGGGGCTTTTATT
IGF1-R	CTCCAGCCTCCTTAGATCACA

**Table 2:** Duration and temperature programs used for the PCR.

Program	Step 1	Step 2	Step 3	Step 4
Temperature (°C)	25	37	85	4
Time	10 min	120 min	5 min	∞ (Forever/10 min)

**Table 3:** Patient characteristics.

Variable	Study Group (N = 10)
Age (years)	35.1 ± 7.6
Infertility duration (years)	3.4 ± 2.01
Nulliparous, n (%)	6 (60%)
Baseline FSH (Follicular stimulation hormone) (IU/L)	6.65 ± 1.91
Baseline LH(luteinizng hormone) (IU/L)	6.36 ± 4.03
Baseline E2 (Estradiol) (pmole/mL)	211.4 ± 98.5
Hysteroscopy indication, n	
Endometrial polyp	3
Repeated implantation failure	3
Repeated pregnancy loss	2
Other (previous cesarean section, prolonged infertility)	2
Last endometrial thickness (mm) before transfer	6.94 ± 3.51
Antibiotic treatment, n (%)	
First line (doxycycline)	9 (90%)
Second line (ciprofloxacin + metronidazole)	8 (80%)
IVF (In vitro fertilization) cycles before treatment	4 ± 3.09
IVF cycles after treatment	3.3 ± 2.7
Pregnancy (positive hCG(Human chorionic gonadotropin), n (%)	8 (80%)
Live birth, n (%)	6 (60%)
Pathologic signs of CE (chronic enndometritis) in hysteroscopy, n (%)	
Hyperemia	3 (30%)
Micropolyps	4 (40%)
Focal edema	3 (30%)
Normal pathology	2 (20%)
Not described	2 (20%)

## Data Availability

The original contributions presented in this study are included in the article. Further inquiries can be directed to the corresponding author.

## Abbreviations

ART, assisted reproductive technology; CE, chronic endometritis; DCs, dendritic cells; ET, embryo transfer; E2, Estradiol; FSH, follicular stimulation hormone; HCG, Human chorionic gonadotropin; HPFs, high-power fields; *HOXA10*, Homeobox A10; *IGF-1*, insulin-like growth factor 1; IHC immunohistochemistry; IVF, in vitro fertilization; LH, luteinizing hormone; MUM-1, multiple myeloma oncogene 1; RT-PCR, real-time polymerase chain reaction; RIF, repeated implantation failure; RPL, recurrent pregnancy loss.
